# MRI of Paraspinal Gossypiboma: Look for the Barium Sulfate Filament

**DOI:** 10.5334/jbsr.3145

**Published:** 2023-11-08

**Authors:** Julian Kent Seghers, Tom Oyen, Jan Erik Vandevenne

**Affiliations:** 1 Southern Methodist University, Dallas, US; 2 Ziekenhuis Oost-Limburg (ZOL), Genk, BE; 3 University of Hasselt, Hasselt, BE

**Keywords:** Textiloma, Gossypiboma, MRI, Barium Sulphate, Wire

## Abstract

Gossypiboma is a rare post-surgical complication comprising a retained surgical gauze surrounded by a foreign body reaction. Although usually presenting on magnetic resonance imaging (MRI) with low T1 signal, high central and low peripheral signal on T2, and bandlike peripheral enhancement, MR appearance is often non-specific. The barium sulphate filament within a surgical gauze presents on MR as a curvilinear thread which is dark on both T1 and T2 sequences. Scrutinizing the MR images is critical to identify the filament and to pinpoint the diagnosis of gossypiboma.

**Teaching Point:** A paraspinal mass on postoperative spine MRI should be carefully searched for a hypointense contorted wire (the barium sulfate filament), as it may be the characteristic finding to evocate the diagnosis of gossypiboma.

## Introduction

Surgical gauzes can accidentally be left behind postoperatively causing in 80% of cases a gossypiboma, which refers to a mass within the body that comprises a cotton or synthetic matrix (textiloma) surrounded by a foreign body reaction [1]. Surgical gauzes contain radiopaque barium sulfate filaments to enable detection on fluoroscopy and radiography. The varied presentations on MR imaging make diagnosis of gossypiboma challenging and some are only diagnosed years after surgery, as they mimic more common masses such as seromas, cysts, abscesses, and tumors [2,3]. In this report, a case of paravertebral gossypiboma is reported, diagnosed by identifying the barium sulfate filament on MRI.

## Case History

A 59-year-old female complained of persistent, aggravating pain in the left lower limb attributed to a left L4–L5 extraforaminal disk herniation impinging on the L4 nerve. Symptoms did not resolve after transforaminal steroid injections, prompting surgical intervention resulting in pain relief.

**Figure 1:**
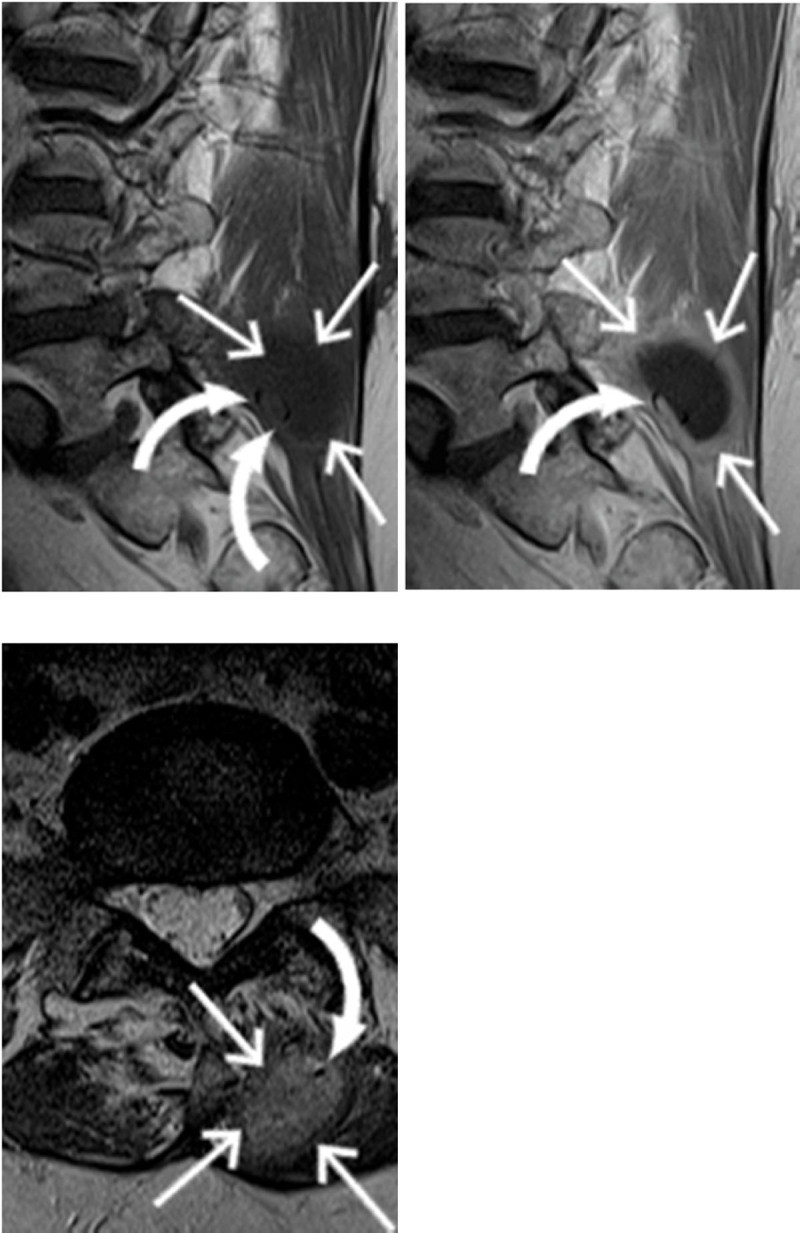
Sagittal T1-weighted MR images before and after intravenous contrast administration. Axial T2-weighted image. Paraspinal mass (arrows) at three months postoperatively interpreted as a postoperative seroma. Retrospectively, the presence of a barium sulfate filament (curved arrows) was noted as a hypointense curvilinear wire.

Three months postoperatively, the patient reported focal discomfort in the lower back. MR imaging showed a well-defined rounded lesion in the paraspinal muscles in the surgical bed (Figure 1). The radiology report suggested a postoperative seroma and the surgeon decided to follow up with the patient.

**Figure 2:**
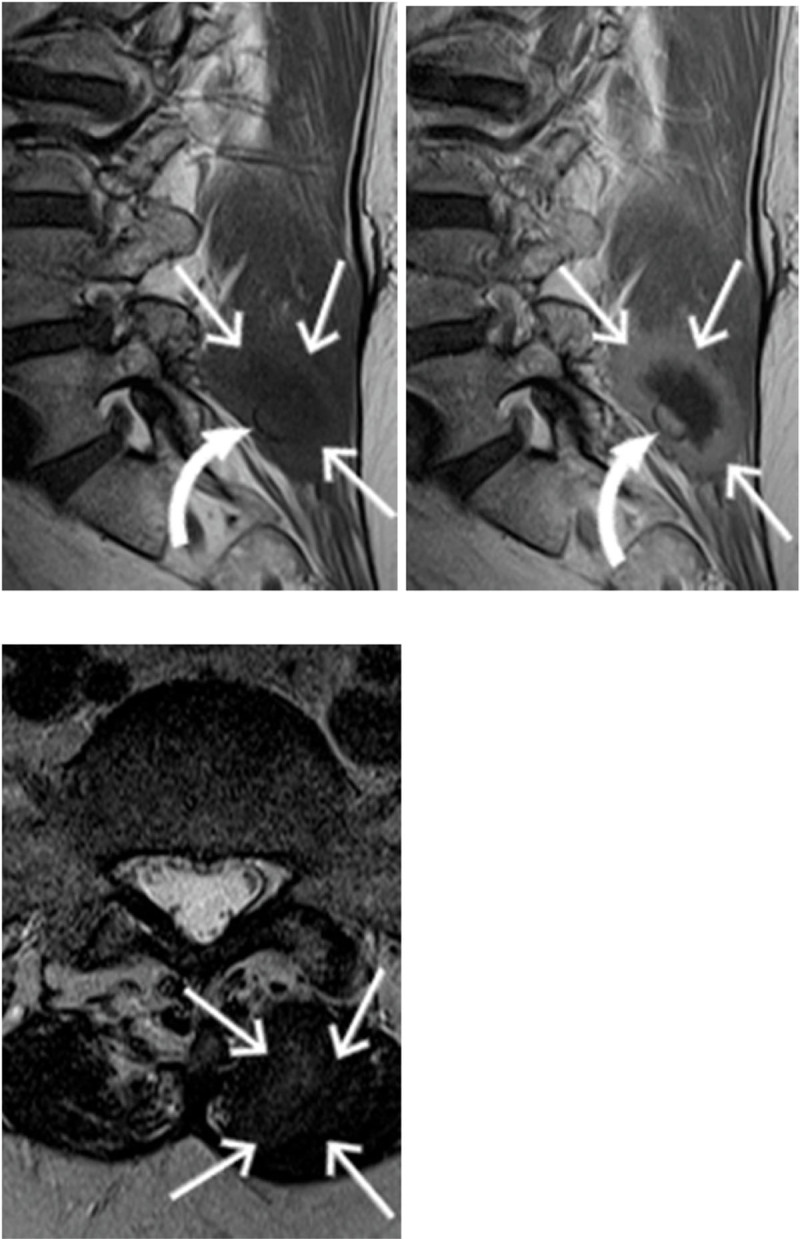
Sagittal T1-weighted MR images before and after intravenous contrast administration. Axial T2-weighted image. Paraspinal mass (arrows) at fourteen months postoperatively. Center of the mass has become hypointense on T2 and the enhancing peripheral wall has thickened. The discovery of a barium sulfate filament (curved arrows) was the characteristic finding to evocate the diagnosis of gossypiboma.

**Figure 3:**
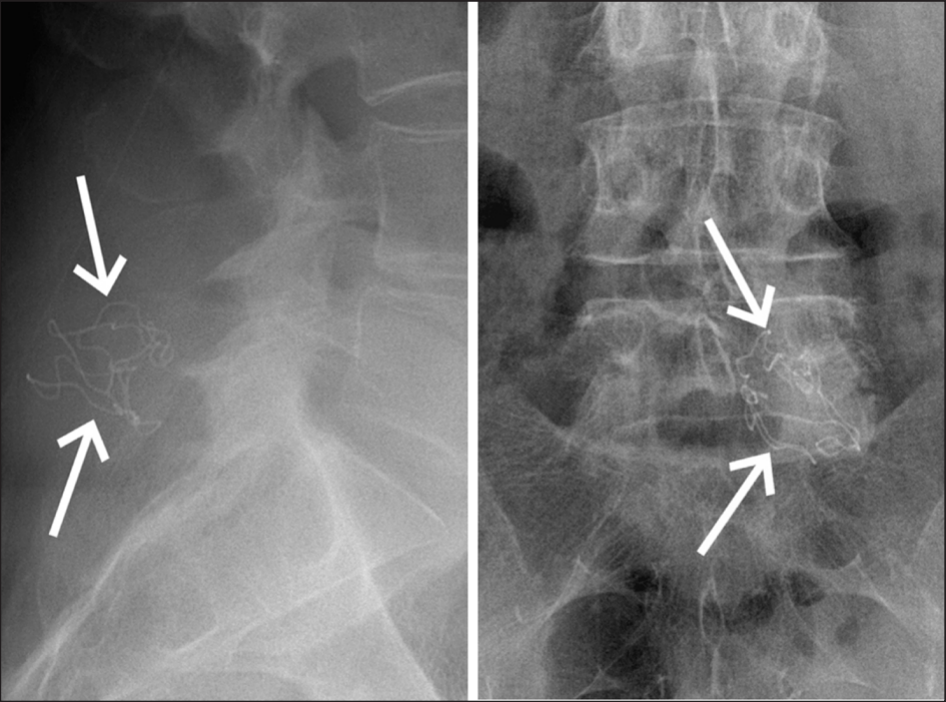
Lateral and frontal radiograph showing the contorted barium sulfate filament confirming the presence of a surgical gauze in the paraspinal soft tissues.

Subsequent MR imaging after 14 months documented a similar mass in the surgical bed although now with homogeneous low signal on T2-weighted imaging and further thickening of the peripheral enhancing wall surrounding the lesion. Additionally, the presence of a intralesional curvilinear low signal structure was noted, prompting the possibility of a gossypiboma from a retained surgical gauze (Figure 2), confirmed on a subsequent radiograph (Figure 3). The mass was surgically removed and composed of inflammatory tissue and residual surgical gauze materials, consistent with a gossypiboma.

## Comments

MR features of gossypibomas typically include low or intermediate signal on T1-weighted sequences, high signal within the central aspect of the lesion and low signal at the peripheral rim on T2-weighted images, and band-like enhancement peripherally [1,2,3]. The nonabsorbable material of the retained surgical foreign bodies may induce either exudative or aseptic granulomatous reactions. Exudative reactions often result in abscess formation and local inflammation, causing patients to become symptomatic in the early postoperative period. However, well-encapsulated aseptic foreign body granulomas may cause no symptoms or only mild non-specific symptoms, explaining them to be undiagnosed or only being discovered years after surgery [3,4]. Thus, there is also a varying MRI presentation of gossypibomas related to the amount of retained fluid and protein.

Detection and awareness of the barium sulfate marker on MR imaging is unique to this case enabling the diagnosis of paraspinal gossypiboma. Barium sulfate filaments are radiopaque, and while readily identified on radiography are difficult to visualize and identify on MRI. Barium sulfate is neither paramagnetic nor magnetic, causing no artifacts on MRI. It also does not contain free protons and therefore does not generate an MR signal, causing it to appear dark on both T1 and T2 sequences, as in our patient [5]. The barium sulfate filament is also thin at 1–2 mm, less than the typical slice thickness on routine MR examinations.

## Conclusion

Despite an often varied clinical and MRI presentation, paravertebral gossypibomas typically appear dark on T1 with high central and low peripheral signal on T2-weighted imaging, along with peripheral, broad bandlike enhancement. These lesions may be misdiagnosed for years as seromas, abscesses, or tumors. Radiologists should be aware of this rare entity and a careful search detecting the hypointense, curvilinear barium sulfate filament may be the trigger for the correct diagnosis.
